# [Retracted] MicroRNA-493-5p promotes apoptosis and suppresses proliferation and invasion in liver cancer cells by targeting VAMP2

**DOI:** 10.3892/ijmm.2025.5711

**Published:** 2025-12-08

**Authors:** Guannan Wang, Xiaosan Fang, Meng Han, Xiaoming Wang, Qiang Huang

Int J Mol Med 41: 1740-1748, 2018; DOI: 10.3892/ijmm.2018.3358

Following the publication of this paper, it was drawn to the Editor's attention by a concerned reader that the 'Normal ctrl' and 'mimic ctrl' data panels shown for the flow cytometry experiments in Fig. 3B on p. 1743 were strikingly similar to data panels that had already been published in an article in the journal *Molecular and Cellular Biochemistry* which had been written by different authors at different research institutes.

Owing to the fact that the contentious data in the above article were found to be strikingly similar to data that had already been published elsewhere, the Editor of *International Journal of Molecular Medicine* has decided that this paper should be retracted from the Journal. The authors were asked for an explanation to account for these concerns, but the Editorial Office did not receive a reply. The Editor apologizes to the readership for any inconvenience caused.

